# Corticosteroid therapy in regressive autism: Preliminary findings from a retrospective study

**DOI:** 10.1186/1741-7015-12-79

**Published:** 2014-05-15

**Authors:** Sailaja Golla, John A Sweeney

**Affiliations:** 1 Division of Pediatric Neurology, Neurodevelopmental Pediatrics, UT Southwestern and Children's Medical Center at Dallas, Dallas, USA; 2 Center for Autism and Developmental Disabilities, UT Southwestern and Children’s Medical Center at Dallas, Dallas, USA

**Keywords:** Autism, Regression, Corticosteroids, FMAER, Language, Behavior

## Abstract

Some children with autism spectrum disorders (ASD; 15% to 30% of patients) show a significant and persistent regression in speech and social function during early childhood. There are no established treatments for the regressive symptoms. However, there are some known causes of this type of regression, such as Rett syndrome and Landau-Kleffner syndrome (LKS). In LKS, steroids have been used as a treatment. Some evidence suggests an autoimmune contribution to the pathophysiology of autism (Chez MG, Guido-Estrada N: Immune therapy in autism: historical experience and future directions with immunomodulatory therapy. *Neurotherapeutics* 2010, 7:293–301, Wasilewska J, Kaczmarski M, Stasiak-Barmuta A, Tobolczyk J, Kowalewska E: Low serum IgA and increased expression of CD23 on B lymphocytes in peripheral blood in children with regressive autism aged 3-6 years old. *Arch Med Sci* 2012, 8:324–331, Stefanatos G: Changing perspectives on Landau-Kleffner syndrome. *Clin Neuropsychol* 2011, 25:963–988), raising the possibility that steroids might be a useful therapy for regression in ASD. A retrospective study published in BMC Neurology by Duffy *et al*. (Duffy, *et al*: Corticosteroid therapy in regressive autism: A retrospective study of effects on the Frequency Modulated Auditory Evoked Response (FMAER), language, and behavior. *BMC Neurol* 2014, 14:70) reviewed 20 steroid treated R-ASD (STAR) patients and 24 ASD control patients not treated with steroids (NSA). Improvements in clinical function and in a neurophysiological biomarker were seen in the steroid-treated children pre- to post-prednisolone treatment. This research provides a rationale for a randomized trial with steroid therapy to determine the longer term benefits and complications of steroids in this population.

Please see related article http://www.biomedcentral.com/1471-2377/14/70/abstract.

## Background

Regression is a highly distinctive feature in some patients with autism characterized by sustained loss of normally developing language and social skills around the age of 15 to 30 months [1]. Regressive features in patients with autism spectrum disorders (ASD) are estimated to occur in as many as 32% of cases, with a mean age of presentation of 1.8 years [2]. It remains to be determined whether this is a biologically distinct form of autism, and currently there are few treatment options to stop or reverse the regression process. An immunologic basis for the pathogenesis of some forms of autism has been hypothesized [3,4], and immune system dysfunction, especially involving B-cell activation, has been implicated as a potential cause for the onset of regression in ASD [5]. In this case, steroids could have a role in arresting progression of such symptoms. They have been successfully used in related epileptic syndromes such as Landau-Kleffner syndrome [LKS] which is associated with regression of speech and acquired epileptiform aphasia [6]. Knowledge of benefits from steroid use in treating regressive autism is limited to small studies. In one case study, a previously developmentally normal child who had autistic regression was treated with low dose steroids with improved speech and developmental milestones in the setting of an autoimmune spectrum of lymphoproliferation and autoimmune hemolytic anemia [7].

In a retrospective case-control study by Duffy *et al*. [8], the authors summarize their single-center experience with steroid treatment using an innovative EEG/ERP biomarker strategy monitoring the frequency modulated auditory evoked response (FMAER).

## Detection of response

The rationale and procedures for evaluating the FMAER have previously been described [9]. In brief, evoked responses in the auditory cortex to rapid auditory stimulus frequency modulation can be used to monitor neurophysiological processes that are important for phoneme detection and language processing. It is a promising, objective diagnostic tool for detecting sensory processing abnormalities in the auditory cortex of functional significance, and a potential biomarker for identifying subgroups of patients for novel treatments and for tracking effects of novel therapies. In this retrospective study, the investigators selected 20 target group children (STAR) with a documented regressive course of autism who had received steroid therapy (prednisolone) and 24 untreated comparison subjects from an existing clinical database [8]. Change in clinical status and FMAER after treatment were assessed and compared with change in controls (NA). The STAR group showed a significant clinical improvement after steroids in receptive and expressive language skills and behavioral manifestations of autism. Improvement in the FMAER was also seen. Almost all patients in the STAR group had steroid related side effects, the majority of which were mild and reversible.

## Potential for steroid therapy?

This study offers promising new data regarding the potential of steroid therapy in regressive autism. While supporting the rationale for a randomized multi-site trial to establish efficacy of the therapy, it is important to recognize that this is a retrospective study involving a small group of children [8]. Factors impacting decision to treat in the clinic might have influenced study findings. There are a significant number of side effects associated with steroids that need to be considered in evaluating risks as well as benefits of the treatment. Treated patients may need close monitoring, including frequent checks of blood pressure, blood glucose, weight gain and potential behavioral effects including mood change and psychosis. A longer trial with consistent follow-up would better establish beneficial changes in language and behavior. Since steroids have been successfully used in multiple neurologic disorders including autoimmune neurologic disorders, epilepsy, muscular dystrophies and encephalitis, neurologists are familiar with their use which could facilitate their use for regressive autism if efficacy can be established.

## Conclusions

This study showing improved function over time in steroid-treated children with regressive autism provides a rationale for a randomized trial of steroid treatment in this population, for which no current treatments are available. Corticosteroids have numerous adverse effects, some of which can lead to potentially serious complications, so a detailed cost-benefit analysis of this treatment in a randomized trial is needed to establish that benefits outweigh risks in this population. This study also provides preliminary support for the use of FMAER as an urgently needed and objective biomarker for evaluating treatment effects on brain function for children with autism, at least for those with regression of language skills.

## Abbreviations

ASD: autism spectrum disorder; CLSQ: Clinical language status questionnaire; EEG: electroencephalogram/electroencephalographic; FMAER: 4 Hz frequency modulated auditory evoked response; LKS: Landau-Kleffner syndrome; NSA: non-steroid treated group; R-ASD: regressive ASD; STAR: steroid treated Autism with regression group; STG: superior temporal gyrus/gyri.

## Competing interests

Dr. Golla has no competing interests. Dr Sweeney consults to Takeda, Lilly, BMS and Roche.

## Authors’ contributions

Both authors contributed to conception of the article, were involved in editing and revision of the manuscript, and agreed to its publication.
